# Mucosal-associated invariant T (MAIT) cells are reduced and dysfunctional in acute melioidosis

**DOI:** 10.1038/s41598-026-57890-8

**Published:** 2026-06-15

**Authors:** Fazle Rabbi Chowdhury, Martha Zewdie, Priyanka Abraham, Srija Moulik, Hadiza Adebowale, Jennifer Hill, Md Robed Amin, Lovely Barai, Md Rokibul Hasan, Ayako Kurioka, Patpong Rongkard, Direk Limmathurotsakul, Nicholas P. J. Day, Parinya Chamnan, Paul Klenerman, Chris B. Willberg, Barbara Kronsteiner, Susanna J. Dunachie

**Affiliations:** 1 Peter Medawar Building for Pathogen Research, University of Oxford, 3 South Parks Road, Oxford, OX1 3SY UK; 2 NDM Centre for Global Health Research, University of Oxford, Oxford, UK; 3 Department of Internal Medicine, Bangladesh Medical University, Dhaka, Bangladesh; 4 Mahidol-Oxford Tropical Medicine Research Unit, Mahidol University, Bangkok, Thailand; 5 Department of Medicine, Dhaka Medical College, Dhaka, Bangladesh; 6 Directorate General of Health Services, Dhaka, Bangladesh; 7 Department of Microbiology, BIRDEM General Hospital, Dhaka, Bangladesh; 8 Department of Tropical Hygiene, Faculty of Tropical Medicine, Mahidol University, Bangkok, Thailand; 9 Cardiometabolic Research Group, Sunpasitthiprasong Hospital, Ubon Ratchathani, Thailand; 10 Oxford University Hospitals NHS Foundation Trust, Oxford, UK; 11 Translational Gastroenterology Unit, University of Oxford, Oxford, UK; 12 NIHR Oxford Biomedical Research Centre, University of Oxford, Oxford, UK

**Keywords:** Mucosal associated invariant T cells, Sepsis, Melioidosis, Diabetes, Diseases, Immunology, Microbiology

## Abstract

*Burkholderia pseudomallei* (BP), the causative agent of melioidosis, is a major cause of sepsis in Southeast Asia, especially in people with diabetes mellitus (DM). The role of Mucosal-associated invariant T (MAIT) cells; innate-like T cells important for antibacterial immunity; in melioidosis is unknown. We measured MAIT cell activation by BP in vitro using co-culture assays with THP-1 cells, and evaluated MAIT cell frequency, activation, and function ex vivo in an observational cohort (n = 120) of melioidosis patients and endemic controls with and without DM in Thailand. We show that BP induces IFN-γ secretion by MAIT cells in a cytokine dependent manner. In acute melioidosis, circulating MAIT cells, particularly the double-negative (DN) subset, were significantly reduced, and highly activated but dysfunctional, with reduced IFN-γ responses to BP and *E. coli* which were restored upon recovery. Among acute patients, non-survivors showed lower granzyme B and IFN-γ expression. Acute melioidosis patients with DM co-morbidity exhibited reduced DN MAIT cell frequency and responses to *E. coli* compared to non-DM patients. Overall, the frequency and function of MAIT cells is impaired during acute melioidosis, especially in patients with DM, indicating a key role for these cells in antibacterial defence and disease susceptibility.

**Supplementary Information:**

The online version contains supplementary material available at https://doi.org/10.1038/s41598-026-57890-8.

## Introduction

Sepsis is a life-threatening organ dysfunction caused by a dysregulated host response to infection^1^ resulting in millions of deaths each year^2,3^. Bacterial infections are responsible for a major proportion of sepsis cases in the world, and among them Gram-negative bacteria are the main culprit^4,5^. Some of the commonest causative Gram-negative organisms are, *Escherichia coli*, *Klebsiella pneumoniae, Pseudomonas aeruginosa* and *Salmonella enterica* serotype Typhi^6^. In addition, the Gram-negative bacterium *Burkholderia pseudomallei* (BP) which causes the neglected tropical disease melioidosis is an important cause of adult sepsis in Asia^4,5^. With an estimated incidence of 165,000 annual cases worldwide^7^ and an in-hospital case fatality rate of up to 50% in some regions of Thailand^8^, melioidosis causes a significant burden in endemic regions. Several risk factors including diabetes mellitus (DM), chronic kidney and lung disease, malignancy and excessive alcohol use are associated with melioidosis^9^. DM constitutes the biggest risk factor with a 12-fold increased risk of melioidosis, and over 50% of melioidosis patients have this metabolic disease^10^. We and others have previously demonstrated a role for T cells^11^ and natural killer (NK) cells^12,13^ in survival from melioidosis in humans, as well as differences in immunological pathways associated with survival in patients with DM co-morbidity^12^. Recent studies in mouse models point towards an important contribution of unconventional T cells to protection. In the absence of invariant NKT (iNKT) cells, survival time was reduced in *B. pseudomallei* infected BALB/c mice, and early activation of this cell subset contributed to bacterial clearance and enhanced survival^14^. In a C57B6/J mouse model of pulmonary melioidosis the presence of γδT cells was important for survival via a reduction of neutrophil-induced inflammation in the lungs^15^.

Mucosal-associated invariant T (MAIT) cells are another unconventional T cell subset with both innate and adaptive properties^16^. This cell population plays an important role in human immune responses to bacterial and viral infections^17^. MAIT cells are abundant in the gut, liver, lung and blood, comprising 1–10% of peripheral blood T lymphocytes^18^. In humans, MAIT cells express a conserved invariant T cell receptor (TCR) α-chain (Vα7.2-Jα33) and are defined by their restriction to the non-classical MHC class I-related (MR-1) molecule^19,20^. Their natural ligands are derived from folic acid (vitamin B9) and from an intermediate in the microbial biosynthesis of riboflavin (vitamin B2)^21^. Therefore, organisms which synthesise riboflavin precursors (Gram-negative bacteria, Yeasts) can stimulate MAIT cells in an MR-1-dependent manner^20^. MAIT cells can also be activated by cytokines in a TCR-independent manner. Notably, IL-12 and IL-18 are potent inducers of MAIT effector functions^22^. Established mouse models demonstrate a beneficial role of MAIT cells in immune defence against several bacterial pathogens including *K. pneumoniae*^23^, *Francisella tularensis*^24^, *Legionella longbeachae*^25^ and extraintestinal pathogenic *E. coli*^26^. In humans with severe sepsis and septic shock, an early reduction of MAIT cell numbers can be observed compared to healthy controls ^26,27^. The role of MAIT cells in melioidosis has not been explored before. In this study we characterise the ability of BP to activate MAIT cells and how MAIT cell frequency and function is affected in a cohort of acute melioidosis patients from Thailand. We further assess the contribution of DM co-morbidity on the properties of these important effector cells.

## Results

### *B. pseudomallei activates MAIT cells *in vitro* in a cytokine dependent manner*

**Fig. 1:**
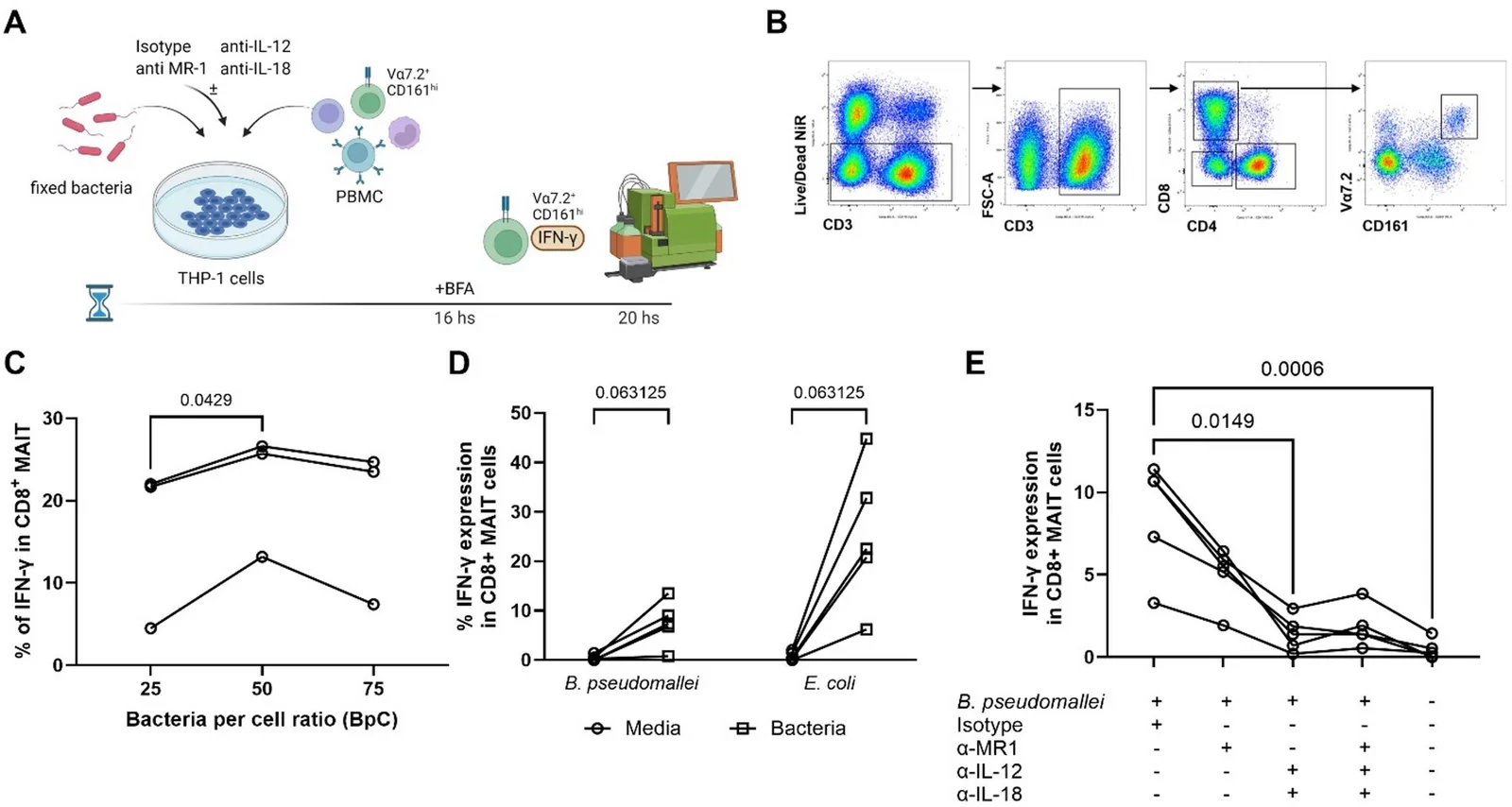
*B. pseudomallei* activates MAIT cells in a cytokine dependent manner. (**A**) Schematic representation of experimental setup. A MAIT cell activation assay was performed by culturing human peripheral blood mononuclear cells (PBMC), THP-1 cells and paraformaldehyde-fixed bacteria together for 20 h followed by intracellular cytokine staining by flow cytometry. BFA = brefeldin A. (**B**) Gating strategy for identification of MAIT cells: within the CD3+ live cell gate different T cell populations were identified based on CD4 and CD8 expression. Subsequent gating for CD161^hi^ and Vα 7.2 + identifies MAIT cells in the respective T cell populations. (**C**) Proportion of CD8+ MAIT cells expressing IFN-γ following co-culture with THP-1 cells and fixed *B. pseudomallei* at different bacteria per cell ratio (BpC, n = 3 healthy individuals). (D) Paired analysis of IFN-γ expression upon culture with THP-1 cells and fixed bacteria (*B. pseudomallei* 50 BpC; *E. coli* 30 BpC) compared to respective media control (n = 5 healthy individuals). (E) Blocking experiment assessing the role of cytokines and TCR activation (n = 5 healthy individuals). MAIT cell induced IFN-γ expression was measured upon stimulation with THP-1 cells and fixed *B. pseudomallei* (50 BpC) in the presence or absence of blocking antibodies against MR-1, IL-12, IL-18 and an isotype control. A two-tailed *p* value of < 0.05 was considered statistically significant. Exact *p* values < 0.1 are displayed on graphs. The data are displayed as spaghetti plots. Paired comparisons were analysed by Wilcoxon rank test for two groups and Friedman test followed by Dunn’s multiple comparison for greater than two groups.

We first assessed the ability of BP to induce IFN-γ secretion from MAIT cells in a previously established co-culture assay^28,29^ using healthy PBMC and THP-1 cells incubated with PFA-fixed BP at 25, 50 and 75 bacteria per cell (BpC) (Fig. 1A). MAIT cells were phenotypically characterised by the expression of Vα7.2 and high expression of CD161 in T cell subsets (Fig. 1B). For this analysis we focused on MAIT cells within the CD8+ T cell subset as this constitutes the majority of MAIT cells in human blood^17,18^. BP induced IFN-γ secretion from circulating CD8+ MAIT cells, with the strongest induction of cytokine expression at BpC 50 (50 BP per THP1 cell). Incubation with 50 BP per THP1 cell was used in all subsequent experiments (Fig. 1C). In comparison to *E. coli,* used at the previously optimised dose of BpC 30^24^, BP induced three-fold lower levels of IFN-γ expression in CD8+ MAIT cells (Fig. 1D). Notably, secretion of IL-12 by THP-1 cells in response to fixed *E.coli* was threefold higher compared to that induced by fixed BP (*E.coli*: 2500 pg/ml, BP: 782 pg/ml). Blocking of both IL-12 and IL-18 during the incubation of BP with THP-1 cells completely abrogated IFN-γ expression (median: 1.4, IQR: 0.5–2.4% IFN-γ positive) compared to the isotype control (median: 10.7, IQR: 5.3–11.1% IFN-γ positive, *p* = 0.0149), indicating that IFN-γ production was predominantly cytokine-dependent. Blocking of MR-1 only marginally reduced cytokine production, and there was no synergistic effect of blocking both cytokines and MR-1 in this culture system (Fig. 1E). This demonstrates that BP is able to activate MAIT cells in a predominantly cytokine-dependent manner at the assessed timepoint.

### Circulating MAIT cells are reduced and dysfunctional in acute melioidosis

**Fig. 2:**
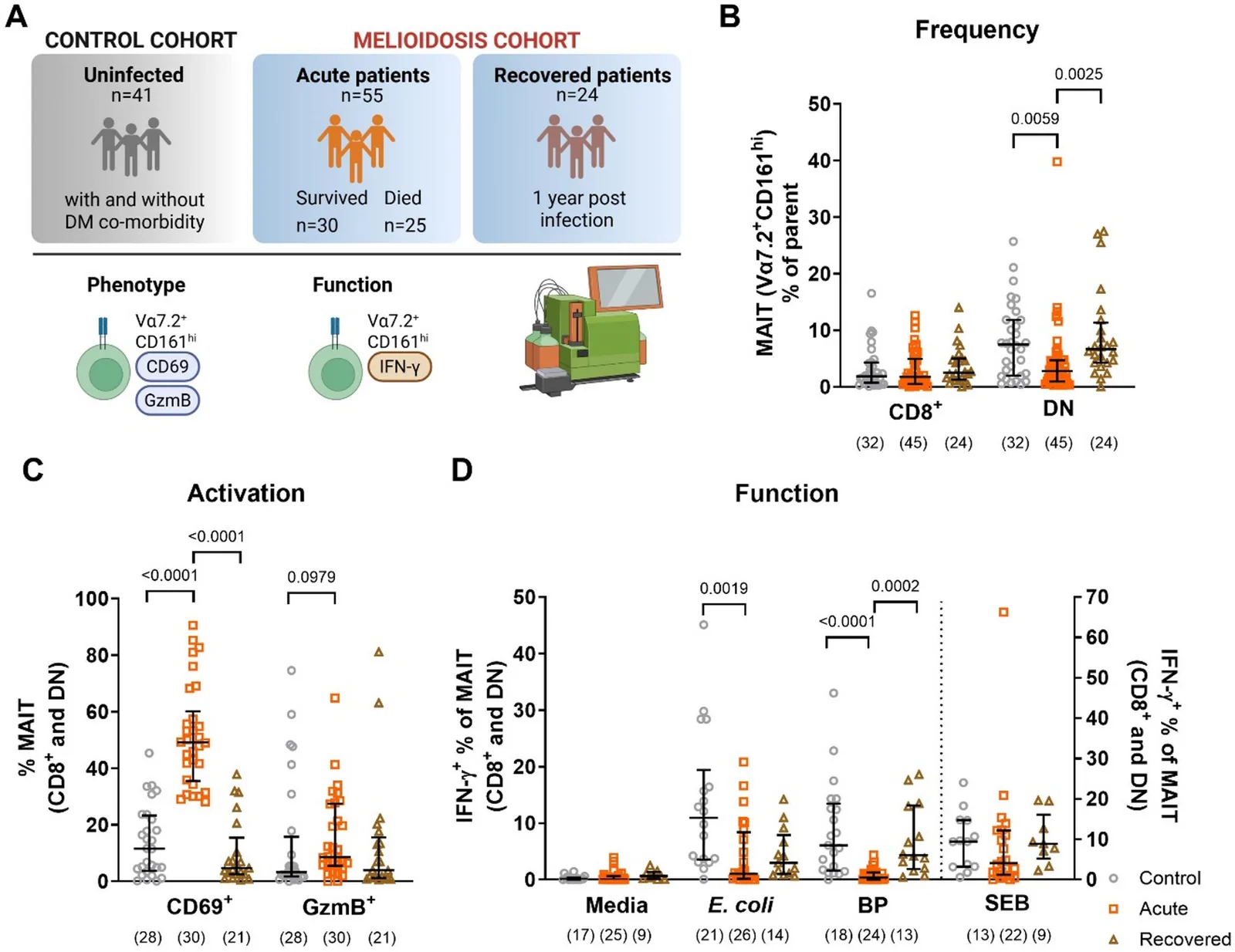
Circulating MAIT cells are reduced, activated and dysfunctional in acute melioidosis. (**A**) Schematic representation of study design. PBMC from acute and recovered melioidosis patients alongside healthy endemic controls (uninfected) were analysed by flow cytometry for MAIT cell frequency, activation (CD69) and cytotoxic granules (GzmB) in MAIT cells ex vivo as well as in vitro IFN-γ expression following stimulation with fixed bacteria. (**B**) Frequency of MAIT cells within the CD8+ and DN T-cell population, (**C**) expression of CD69 and GzmB in CD8+ and DN MAIT cells and (**D**) expression of IFN-γ in MAIT cells upon in vitro stimulation with fixed *E. coli*, *B. pseudomallei* (BP), staphylococcal enterotoxin B (SEB) and media were compared between controls, acute melioidosis patients and those who had recovered from melioidosis (1 year post admission). Grey circles: controls, orange squares: acute patients, brown triangles: recovered patients. The number of biological replicates per group is given in brackets under graphs. A two-tailed *p* value of < 0.05 was considered statistically significant. Exact *p* values < 0.1 are displayed on graphs. The data are displayed as median and interquartile range. The three groups were compared using Kruskal Wallis test followed by Dunn’s multiple comparison. DM = diabetes mellitus.

Having demonstrated that MAIT cells are activated by BP in vitro we next sought to study the frequency, activation and function of MAIT cells ex vivo in the context of acute melioidosis infection. We used samples from a cohort of acute and recovered melioidosis patients recruited in Thailand between 2012 and 2017. Individuals with and without DM from the same region were used as endemic controls (Fig. 2A). Acute melioidosis patients were slightly but significantly older (median 59, IQR 51–65 years, *p* = 0.016) than recovered patients (median 53, IQR 45–61 years) and healthy controls (median 52, IQR 48–61 years) (Table 1). There were no measured differences in sex, the proportion of individuals with DM, or history of renal impairment between groups (Table 1). We found a 2.7-fold reduction in frequency of circulating MAIT cells within the double negative (DN) T cell compartment in acute melioidosis cases (median 2.8, IQR 0.9–4.7%) compared with healthy controls (median 7.5, IQR 2.0–11.8%, *p* = 0.0059) (Fig. 2B**).** Importantly, one year after infection the frequency of DN MAIT cells in recovered patients (median 6.7, IQR 4.2–11.3%) returned to levels comparable to healthy controls (Fig. 2B). The same effect was observed when comparing the frequency of MAIT cells in the overall live cell compartment (Supplementary Fig. 1). In contrast, the frequency of CD8+ MAIT cells remained unaffected during acute disease.

**Table 1:** Participant characteristics for melioidosis cases (acute and recovered) and endemic controls.

	Melioidosis			
	Acute	Recovered	Control	*p* value*
	N = 55	N = 24	N = 41	
Age				0.016
Median age	59	53	52	
Age range	33–84	25–88	33–73	
Interquartile range	51–65	45–61	48–61	
Sex, n (%)				0.45
Female	16 (29%)	8 (33%)	17 (41%)	
Male	39 (71%)	16 (67%)	24 (59%)	
28-day mortality, n (%)				NA
Survived	30 (55%)	24 (100%)	41 (100%)	
Died	25 (45%)	0 (0%)	0 (0%)	
Co-morbidities, n (%)				
Diabetes	31 (56%)	12 (50%)	22 (54%)	0.87
History of renal impairment	11(23%)	5 (21%)	0 (NA%)	> 0.99

*Pearson Chi-Squared test; Kruskal–Wallis rank sum test; Fisher’s exact test.

Next, we assessed activation and functional properties of MAIT cells. Due to the low numbers of MAIT cells in acute disease we combined CD8+ and DN MAIT cell compartments in order to increase events for detection of downstream parameters by flow cytometry and to maximise the number of biological replicates per group. MAIT cells (CD8+ and DN combined) were highly activated (CD69+) in acute melioidosis (median 49.1, IQR 35.5–60.1%) compared with healthy controls (median 11.6, IQR 3.7–23.2%, *p* < 0.0001) and recovered patients (median 4.7, IQR 2.5–15.4%, *p* < 0.0001). MAIT cells displayed higher GzmB expression in acute disease (median 8.6, IQR 5.5–27.5%) compared with healthy controls, (median 3.3, IQR 1.7–15.8%) although this difference was not statistically significant (*p* = 0.0979) (Fig. 2C). Furthermore, IFN-γ production by MAIT cells from acute melioidosis patients following in vitro stimulation was abrogated compared with healthy controls. Responses to both BP (median, IQR: Acute 0.4, 0–1.2%; Healthy 6.1, 1.6–13.5%, *p* < 0.0001) and *E. coli* (median, IQR: Acute 1.0, 0.2–8.4%; Healthy 11.0, 3.6–19.4%, *p* = 0.0019) were significantly reduced. Upon recovery, IFN- γ expression in response to BP returned to levels comparable with the control group but this was not the case for *E. coli* (Fig. 2D, Supplementary Fig. 2). We obtained similar results when analysing CD8+ and DN MAIT cells separately (Supplementary Fig. 3A, B) which demonstrates that impaired MAIT function in acute melioidosis was not specific to any one subset. To assess whether a reduced capacity for IFN-γ expression in MAIT cells is a particular feature of melioidosis or a more common effect in sepsis we measured MAIT cell IFN-γ expression in a small cohort of sepsis patients with different disease aetiology in Bangladesh. MAIT cell IFN-γ expression upon stimulation with the respective causative pathogen was significantly impaired in patients infected with *K. pneumoniae* and *E. coli* (Supplementary Fig. 4B, C) and a trend for reduced levels was observed in those infected with *S.* Typhi (Supplementary Fig. 4A) compared with healthy controls. Overall, our data highlights depletion of MAIT cells in the DN T cell compartment in acute melioidosis, and shows that despite being highly activated in acute disease, MAIT cells are limited in their ability to produce IFN-γ in response to bacterial stimulation in vitro.

### Impaired GzmB and IFN-γ expression in MAIT cells of non-survivors

**Fig. 3:**
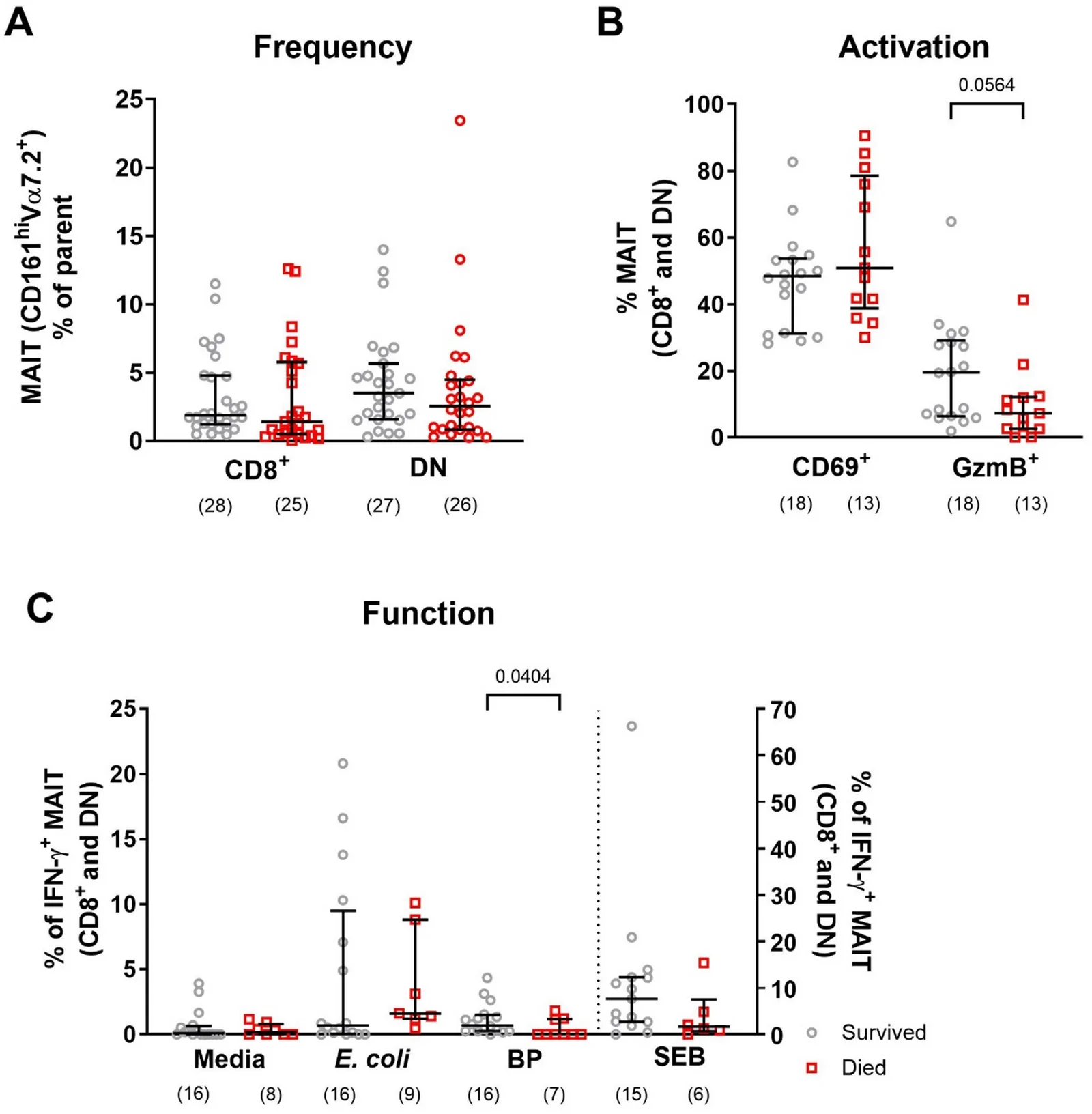
Functional changes were observed in circulating MAIT cells in acute melioidosis survivors compared to those who died. (**A**) Frequency of MAIT cells within the CD8+ and DN T-cell population, (**B**) expression of CD69 and GzmB in CD8+ and DN MAIT cells and (**C**) expression of IFN-γ in MAIT cells following in vitro stimulation with fixed *E. coli*, *B. pseudomallei* (BP), staphylococcal enterotoxin B (SEB) and media were compared between acute melioidosis patients who died (red squares) within 28 days of admission and those who survived (grey circles). The number of biological replicates per group is given in brackets under graphs. A two-tailed *p* value of < 0.05 was considered statistically significant. Exact *p* values < 0.1 are displayed on graphs. The data are displayed as median and interquartile range. The two groups were compared using Mann Whitney U test.

We have previously shown that IFN-γ secretion by conventional T cells is impaired in acute melioidosis patients who died compared to those who survived^11^. To address whether a similar effect is observed for MAIT cells we stratified the acute melioidosis cohort based on clinical outcome (Supplementary Table 2). While frequency and activation (CD69 +) of MAIT cells were comparable in patients who survived and those who died within 28-days of hospital admission (Fig. 3A, B), GzmB expression (Fig. 3B) and IFN-γ production (Fig. 3C) in response to BP were reduced in non-survivors (GzmB median, IQR: Survived 19.5, 6.3–29.2%; Died 7.3, 2.6–12.2%, *p* = 0.0564; IFN-γ median, IQR: Survived 0.7, 0.3–1.5%; Died 0.0, 0.0–1.2%, *p* = 0.0404). This shows a further impairment of MAIT cell function in patients with fatal outcome.

## MAIT cell frequency and function are reduced in acute melioidosis patients with DM

**Fig. 4:**
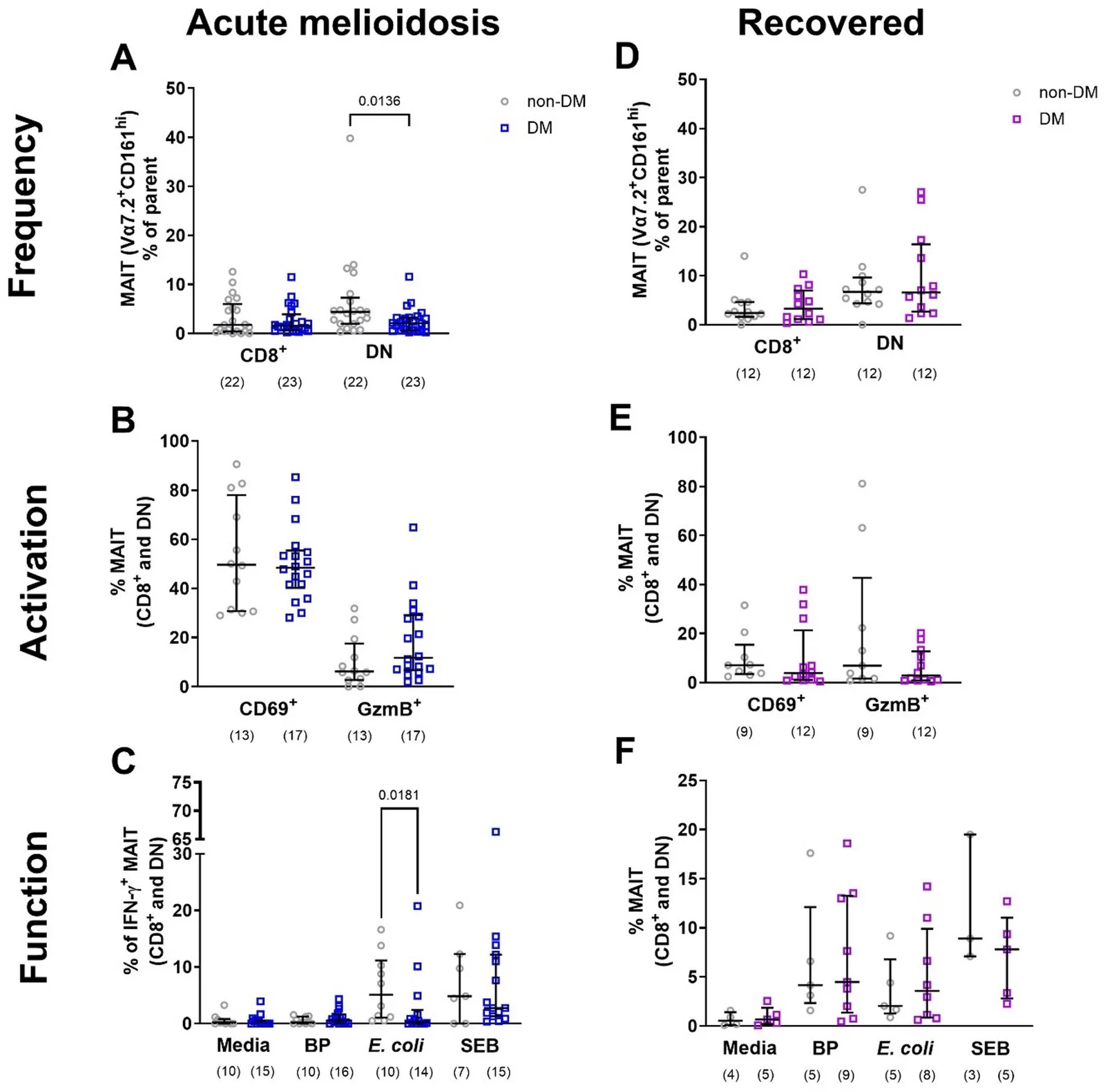
Diabetes drives MAIT cell changes in acute melioidosis. The acute and recovered melioidosis cohorts were split according to diabetes mellitus (DM) status. (**A**, **D**) The frequency of MAIT cells within the CD8+ and DN T-cell population, (**B**, **E**) expression of CD69 and GzmB in CD8+ and DN MAIT cells and (**C**, **F**) expression of IFN-γ in MAIT cells upon in vitro stimulation with fixed *E. coli*, *B. pseudomallei* (BP), staphylococcal enterotoxin B (SEB) and media were compared between the non-DM (circles) and DM (squares) group. The number of biological replicates per group is given in brackets under graphs. A two-tailed *p* value of < 0.05 was considered statistically significant. Exact *p* values < 0.1 are displayed on graphs. The data are displayed as median and interquartile range. The two groups were compared using Mann Whitney U test.

Since DM is an independent risk factor for melioidosis, we compared the frequency and activation of MAIT cells in acute and recovered melioidosis patients between those with and without DM (Supplementary Table 3). Acute melioidosis patients with DM showed a 50% reduction in the proportion of MAIT cells within the DN T cell compartment (median 2.1, IQR 0.6–3.2%) compared with the non-DM group (median 4.4, IQR 2.0–7.3%, *p* = 0.0136). No difference was observed for the CD8+ MAIT cell subset (Fig. 4A). MAIT cell expression of CD69 and GzmB was not significantly different in acute patients with DM compared to those without but we note that GzmB expression is slightly elevated in the DM group (Fig. 4B). Importantly, IFN-γ production by MAIT cells in response to *E. coli* was significantly impaired in acute melioidosis patients with DM (median 0.3, IQR 0.0–2.4%) compared to those without DM (median 5.1, IQR 1.0–11.2%, *p* = 0.0181, Fig. 4C). In contrast. recovered patients did not show differences in cell frequency, activation or function based on DM status (Fig. 4D–F) indicating that MAIT cell dysfunction in DM is context dependent.

### Association of MAIT cell features with clinical parameters

In order to assess the combined impact of clinical and demographic factors on MAIT cell frequency and activation we performed GLM analysis using age, sex, DM status, 28-day mortality and history of renal impairment as covariates. In line with univariate analysis, we identified DM as being significantly associated with a reduced proportion of MAIT cells within the DN T cell compartment and 28-day mortality being associated with lower expression of GzmB in MAIT cells (Table 2). In contrast, DM was independently associated with significantly higher expression of GzmB in MAIT cells when taking relevant covariates into account. Differences in CD69 expression on MAIT cells were not associated with any of the tested variables.

**Table 2:** Generalized linear model testing the association of MAIT frequency and activation with demographic and clinical parameters.

Variables		Estimates		
Dependent	Predictor	Coef	SE_Coef_	*p* value
DN MAIT % of T cells	Age	0.023	0.015	0.121
	Sex (male)	0.141	0.333	0.672
	28-day Mortality (yes)	− 0.514	0.311	0.098
	Diabetes (yes)	− **0.566**	0.285	**0.047**
	History of renal impairment (yes)	0.574	0.332	0.084
CD69% of MAIT cells	Age	0.006	0.007	0.374
	Sex (male)	0.116	0.126	0.359
	28-day Mortality (yes)	0.116	0.132	0.381
	Diabetes (yes)	− 0.135	0.133	0.313
	History of renal impairment (yes)	0.146	0.132	0.265
GzmB % of MAIT cells	Age	− 0.001	0.024	0.971
	Sex (male)	− 0.185	0.383	0.629
	28-day Mortality (yes)	− **0.854**	0.410	**0.037**
	Diabetes (yes)	**1.070**	0.426	**0.012**
	History of renal impairment (yes)	0.340	0.416	0.413

Significant values are in bold.

Gamma log linked, Coef = Coefficient, SE = standard error.

## Discussion

This is the first study to characterise the MAIT cell response in the context of the neglected tropical disease melioidosis. Here we show that BP efficiently induces IFN-γ secretion by Vα7.2 + CD161hi MAIT cells in an overnight (20 h) co-culture system with THP-1 cells. In our hands, blocking of cytokine signalling using antibodies targeted at the receptors for IL-12 and IL-18 completely abrogated the expression of IFN-γ in MAIT cells with no further reduction achieved by additional blocking of MR-1. Ussher et al^29^ previously demonstrated a time dependent increase in IFN-γ production by MAIT cells stimulated with *E. coli*, with early IFN-γ secretion (5 h) being entirely dependent on MR-1, and late IFN-γ secretion (20 h) dependent on MR-1 in combination with IL-12 and IL-18. Similarly, *C. difficile*-induced IFN-γ production was found to be initially MR-1 dependent, with a more pronounced role for cytokines in driving IFN-γ production and cytotoxic features after longer incubation times^30^. In contrast, our data does not provide evidence for combined action of MR-1 and cytokines in induction of MAIT cell IFN-γ in response to BP after overnight culture, suggesting predominantly TCR-independent activation. The extent to which MAIT cells secrete IFN-γ is dependent on the levels of MR-1 upregulation, TLR stimulation and induction of IL-12 in antigen presenting cells (APC)^31^. *E. faecalis,* which is a weak inducer of IL-12 in THP-1 cells, also induced weaker MAIT IFN-γ responses compared to *E coli*, suggesting that MAIT cell activation and cytokine release are pathogen-specific^22^. In line with this, we show that BP compared with *E. coli* is a weaker inducer of IL-12 secretion by primed THP-1 cells, likely resulting in lower induction of IFN-γ by MAIT cells. It is important to note that BP possesses a diverse array of pathogenicity factors which can activate innate immune sensing and actively contribute to immune evasion^32^. BP is known to possess high lipopolysaccharide O-antigen diversity among strains with different geographical distribution^33^ and impact on M1 macrophage polarization^34^. In vitro infection with live BP activates the inflammasome and induces IL-1ß and IL-18 secretion from primary monocytes, bone marrow derived macrophages, alveolar macrophages and THP1 cells^35–37^. The magnitude of this pro-inflammatory cytokine production is partially dependent on the infected cell type and presence of specific virulence factors including flagellin^36^. Infection with the related avirulent species *B. thailandensis* showed significantly lower induction of IL-1ß and IL-18^35^. In contrast to IL-18, IL-12p70 is only marginally induced in primary monocytes upon BP infection and this does not differ from *B. thailandensis*^35^. Overall, the exact pro-inflammatory cytokine milieu induced by host cells will depend on the presence of specific virulence factors. It is currently unknown to what extent diverse clinical isolates impact on IL-12 and IL-18 secretion by myeloid cells and more work is needed to define this variability and its impact on MAIT cell function in vitro and in vivo.

In a cohort of melioidosis patients in Thailand, we show that MAIT cell frequency is significantly reduced during acute infection, with the cells that are present being highly activated but dysfunctional. We observed dampened IFN-γ production by MAIT cells in response to BP and *E.coli*, with responses returning to normal upon recovery. Within the acute melioidosis group we did not find evidence for differences in MAIT cell frequency and activation based on outcome of infection, but impaired function including reduced GzmB expression and IFN-γ production was noted in patients who died within 28-days of hospital admission.

Our findings align with other studies of MAIT cells in acute bacterial disease. Marked specific depletion of MAIT cells was found in acute non-streptococcal septic patients compared to healthy controls, and this was associated with susceptibility to nosocomial infection^27^. Similar findings have been found in inflammatory and infectious diseases including acute cholecystitis^38^, pulmonary tuberculosis (TB)^39,40^ and scrub typhus^41^. In a recent *S*. Typhi human challenge study, CD8+ MAIT cell frequency was sharply decreased early post-infection in volunteers who developed typhoid disease compared to those resistant to disease^42^. Reduced circulating MAIT numbers in infection could be due to recruitment of the cells into sites of infection, or increased apoptosis in the case of sepsis. As demonstrated and reviewed by Hotchkiss et al^43^, extensive apoptosis of immune cells including T cells, B cells and dendritic cells is a hallmark of sepsis. Zheng et al. have recently shown that MAIT cells are reduced in circulation but enriched in tissue during acute appendicitis^44^. Interestingly, the reduction in MAIT cells in acute melioidosis was specific to the DN T cell compartment and not the CD8+ T cell subset. Notably, DN MAIT cells have been shown to exhibit higher apoptotic gene signatures and they are more prone to activation-induced apoptosis^45^ possibly explaining the cell subset specific depletion observed in our study. In acute bacterial infection, there is evidence of diminished functional capacity of circulating MAIT cells^46^. MAIT cell IFN-γ production has been previously shown to be reduced during active mycobacterial infection^39^, which is in line with our findings. We and others have previously shown that IL-18 and IL-18 binding protein (IL-18BP) are elevated in melioidosis non-survivors^12,47^. Importantly, the presence of IL-18 was essential for protection from melioidosis in a mouse model and this was associated with IL-18 dependent induction of IFN-γ^47,48^. Since cytokine driven activation of MAIT cells is partly mediated by this pro-inflammatory cytokine, it is conceivable that reduced availability of IL-18 due to high levels of IL-18BP in melioidosis patients might result in suboptimal activation of MAIT cells and thereby reduced secretion of IFN-γ which is important for the control of infection. Further studies are needed to provide direct evidence for the protective role of MAIT cells in melioidosis.

We identified specific changes in MAIT cells during acute melioidosis in patients with DM, including reduced proportion of DN MAIT cells, increased GzmB expression and reduced IFN-γ production upon stimulation with bacteria. DM, the major risk factor for melioidosis, is associated with chronic inflammation, reduced T cell function, and dysfunction of the innate immune system; specifically neutrophil and monocyte function^49^. MAIT cells have been previously shown to be reduced in frequency and function in blood and adipose tissue of Type 2 DM (T2DM) patients and obese individuals^50,51^. Magalhaes et al. have shown that MAIT cells from T2DM patients have a pro-inflammatory phenotype characterised by higher levels of IL-17, IL-2, IFN-γ and GzmB compared to healthy individuals when activated with PMA/Ionomycin in vitro^50^. In contrast, MAIT cells were less activated upon specific TCR activation and showed a skewed cytokine response away from IFN-γ and TNF towards an IL-17 biased response in T2DM compared to healthy controls^50^. A subset of IL-17 secreting MAIT cells has also been associated with various chronic inflammatory conditions^52^. Our study shows similar impairment of MAIT cell function in the context of acute bacterial infection with reduced IFN-γ secretion in response to *E. coli* in patients with DM co-morbidity. Although beyond the scope of this study, it will be interesting to follow up the IL-17 bias in the context of melioidosis and DM. Overall, our results point toward specific functional alterations of MAIT cells in people with DM during acute melioidosis infection. MAIT cell function was generally low in acute melioidosis with a further reduction seen in those who died from the disease. Future studies are needed to specifically address the contribution of MAIT cells to disease susceptibility in people with DM and their role in early control of bacterial infections such as melioidosis.

### Limitations

The number of MAIT cells was highly reduced in acute disease therefore analysis of downstream properties of MAIT cells was restricted to samples with enough cells. We characterised MAIT cells by their expression of CD161 and Vα7.2 and did not use 5-OP-RU loaded MR1 tetramers for a more direct identification of MAIT cells. We focussed on the measurement of IFN-γ as the main effector molecule produced by MAIT cells upon activation. Measuring IL-17 especially in the context of DM co-morbidity and local immune responses in the lungs might give further insights into the role of MAIT cells in disease susceptibility and melioidosis pathogenicity. For the in vitro work we only used one single strain of BP, the clinical isolate K96243, which is commonly used in melioidosis research. However, given the geographical and antigenic diversity of BP strains this limits the generalisability of findings as different clinical isolates might induce varying degrees of cytokine production by THP-1 cells and thus differences in magnitude of MAIT cell activation.

## Methods

### Patient recruitment

We used a patient cohort recruited into an observational study of acute melioidosis at Sunpasitthiprasong Hospital, Ubon Ratchathani, Thailand between 2012 and 2017^11,12^. Patients over 18 years with culture-confirmed melioidosis at a median of 5 days after admission to hospital (IQR 4–6 days)^11^ were included in the study. 67% of patients had a diagnosis of DM (defined for the purpose of this study as a past medical history of diabetes and/or a blood glycated haemoglobin (HbA1c) of ≥ 7%)^8,12^. 28-day mortality was determined using hospital mortality records and follow-up telephone calls. Recovered melioidosis patients were seen one year after initial enrolment (week 52). Uninfected individuals with and without DM were recruited as endemic controls. Gram-negative culture-confirmed sepsis cases were recruited as part of the *Aetiology of Fever* study at Dhaka Medical College Hospital (DMCH) and Bangladesh Institute of Research and Rehabilitation in Diabetes, Endocrine and Metabolic Disorders (BIRDEM) Hospitals, Dhaka, Bangladesh. Adult patients of 18 years or more who were admitted with a history of fever ≥ 38 degrees for more than 48 h were included. Acute Gram-negative sepsis patients (defined according to SIRS criteria)^53^ were admitted 1–2 weeks into the course of their illness. Healthy endemic control subjects were recruited from the blood transfusion department of DMCH, and critically ill non-infectious patients (acute stroke, acute pesticide poisoning, diabetic ketoacidosis, severe acute asthma and acute exacerbation of COPD) were enrolled from the medicine department at DMCH. Healthy controls from the UK were recruited as part of the GI Biobank study at the University of Oxford and samples were used for MAIT in vitro assays including blocking experiments.

### Ethical approvals

The ethics committees of the Faculty of Tropical Medicine, Mahidol University (TMEC-12-014 and TMEC-15-046), of Sunpasitthiprasong Hospital, Ubon Ratchathani (018/2555 and 017/2559) and the Oxford Tropical Medicine Research Ethics Committee (OXTREC; 64-11 and 35-15) approved the Thai melioidosis study protocol. The ethics committee of DMC (MEU-DMC/ECC/2015/97), BIRDEM Hospital (BADAS-ERC/EC/17/0127), and OXTREC (OXTREC: 51-16) approved the aetiology of febrile illness study in Dhaka, Bangladesh. Healthy unexposed subjects recruited from Oxford gave written informed consent, and a Research Ethics Committee (16/YH/0247) approved the work. Written informed consent was obtained for all participants enrolled in the study, including for storage and export of samples to the UK. Studies were conducted according to the principles of the Declaration of Helsinki (2008) and the International Conference on Harmonization (ICH) Good Clinical Practice (GCP) guidelines.

### Peripheral blood mononuclear cell isolation

PBMCs were isolated from whole blood within three hours of blood draw by density gradient centrifugation using Lymphoprep (AxisShield, Oslo, Norway) and subsequently cryopreserved in heat-inactivated fetal calf serum (FCS) and 10% (v/v) dimethyl sulfoxide (DMSO). Cells were kept at − 80 °C for short-term storage and then transferred to liquid nitrogen for long-term storage. Cells were thawed at 37 °C and washed in R10 media composed of RPMI 1640 (Sigma, St. Louis, MO, USA) containing 10% heat-inactivated FCS (Life Technologies, Carlsbad, CA, USA), 1% L-glutamine, and 1% penicillin/streptomycin (Sigma-Aldrich, Dorset, UK). Samples were treated with 1 µl/ml benzonase (Merck Millipore, Billerica, MA, USA) in R10 for 30 min in a 37 °C, 5% CO_2_, and 95% humidity incubator and subsequently centrifuged and resuspended in R10. Cells were counted using a haemocytometer with trypan blue staining (Sigma) to assess viability before use in downstream assays.

### THP-1 cell maintenance and propagation

A monocyte cell line was used as antigen presenting cell (APC) in all functional MAIT cell experiments. THP-1 cells (ECACC, UK) were cultured in RPMI 1640 with 10% FCS, 1% L-glutamine, and 1% penicillin/streptomycin, and 0.05% β-mercaptoethanol (all Sigma-Aldrich, UK) at 2 × 10^5^ cells/ml.

### Preparation of fixed bacteria

Bacterial strains of *E.coli* (DH5-α), *S.* Typhi (PF CT18) and *K. pneumoniae* (ATCC 13368) were fixed with 2% paraformaldehyde (PFA) and *B. pseudomallei* (K96243) was fixed with 0.5% PFA before use in MAIT cytokine secretion assays. Bacteria were cultured in LB agar except for *B. pseudomallei* which was cultured using TSB and Ashdown’s medium. For fixation 20–40 ml of cell suspension (from an overnight culture) were spun, fixed with PFA, washed three times in 1 × PBS and resuspended in 1–3 ml of PBS. Colony forming units (CFU)/ml were determined by plating serial dilutions of bacteria prior to fixation.

### MAIT cytokine secretion assay (THP-1 co-culture)

THP-1 cells were plated at a density of 80,000 cells per well in a 96-well round-bottom plate and stimulated with PFA-fixed BP, *E. coli, S.* Typhi and *K. pneumoniae*. Bacteria were used at a bacteria per cell ratio (BpC) of 30 for *E. coli* and 50 for the other bacteria unless otherwise stated in the figure legends. Staphylococcal enterotoxin B (SEB, final concentration: 5 ug/ml) was used as a positive control in all the experiments. PBMC were added to THP-1 cells at 80,000 cells/well and cultured overnight (16 h) at 37 °C, 5% CO_2_ and 95% humidity incubator. Brefeldin A (1:1000 final dilution; 20 µl/well) was added to each well for the last 4 h of incubation. Cells were subsequently stained for flow cytometry analysis.

### Cytokine secretion by THP-1 cells

THP-1 cells were cultured with fixed *E. coli* and *B. pseudomallei* as above but in the absence of PBMC. Supernatants were collected after 20 h and stored at -70ºC until further use. IL-12p40 was measured in cell-free supernatants (diluted twofold) using the IL-12p40 Duoset enzyme linked immunosorbent assay (ELISA) Kit according to manufacturer’s instructions (R&D Systems). Results were obtained as absorbance values (450 nm) using a GloMax Explorer microplate reader (Promega). Concentrations of cytokines were calculated from standard curves generated by fitting 4-parameter logistic regression curves to the values of the standard wells using GraphPad Prism Version 10 (San Diego, CA, USA).

### Flow cytometry staining

For activation and phenotyping experiments, 1 × 10^6^ PBMC were re-suspended in phosphate buffered saline (PBS) and incubated for 20 min at 4 °C with near-infrared live/dead fixable stain (Invitrogen, Carlsbad, CA, USA, 1/500) and fluorochrome-conjugated antibodies against extracellular markers: CD3, CD4, CD8, CD161, TCR Vα7.2, CD69 in the presence of human FcR blocking reagent (Miltenyi Biotec). After washing with PBS, cells were fixed with fixation/permeabilization solution (BD Biosciences, eBioscience) for 15 min at 4 °C, washed with permeabilization buffer (BD Biosciences, eBioscience) followed by incubation with a fluorochrome-conjugated antibody against granzyme B (GzmB) in the presence of FcR blocking reagent for 30 min at 4 °C. After washing with permeabilization buffer, cells were resuspended in PBS and stored at 4 °C until acquisition. For the MAIT cytokine secretion assay, a one-step staining was performed. PBMC (1 × 10^6^) were first incubated with viability dye as described above. After washing in PBS, cells were fixed with 2% PFA for 20 min at 4 °C, then stained with fluorochrome conjugated antibodies: CD3, CD4, CD8, CD161, TCR Vα7.2, IFN-γ, and TNF in permeabilization buffer for 20 min at 4 °C. Finally, cells were washed with permeabilization buffer, resuspended in PBS and acquired on a MACSQuant Analyser 10 (Miltenyi Biotec). The acquisition was performed on the same day for all experiments. The following rules were used for quality control of flow cytometry data: a minimum of 1000 events in the CD3 gate and for downstream analysis of MAIT cells (CD69, GzmB, IFN-γ) a minimum of 50 events in the parent gate. Data analysis was performed with FlowJo Version 10 (FlowJo LLC, Ashland, OR, USA). Details of antibody clones, manufacturer and fluorochromes can be found in the Supplementary Table 1. Staining for TNF was performed in the assay but data are not presented in this study.

### Statistical analysis

Categorical variables were expressed as counts and frequencies and compared using Fisher’s exact test. Non-parametric variables (age, immunological parameters, HbA1c) were expressed as median and interquartile range. For continuous variables, differences between two independent groups were calculated using the Mann–Whitney U test and differences between three independent groups were assessed using Kruskal Wallis test using Dunn’s multiple comparison. For comparisons of paired data Wilcoxon signed rank test was used to compare two groups and Friedman test followed by Dunn’s multiple comparison was used to compare three or more groups. A two-tailed *p* value of < 0.05 was considered statistically significant. To evaluate the association of continuous experimental variables with demographic and clinical characteristics we first assessed the distribution of the outcome variables using descriptive statistics, visual inspection of histograms, and Shapiro–Wilk test. The outcome variables showed a positively skewed distribution, and inspection of residual-versus-fitted plots indicated heteroscedasticity, with increasing residual variance at higher fitted values. Therefore, a generalized linear model (GLM) with Gamma distribution and log link was chosen for the analysis.

Multicollinearity among predictors was assessed using variance inflation factors (VIF). All VIF values were below 2, indicating negligible multicollinearity. A clinically relevant interaction term between renal disease and 28-day mortality was evaluated. This interaction term was not statistically significant and did not improve model fit as demonstrated by negligible changes in Akaike’s Information Criterion (AIC) and log-likelihood values compared to the main-effects model. Given the lack of evidence for effect modification and the relatively small sample size (n = 26–38) the simpler main-effects model was retained. Statistical analysis was performed using SPSS Version 29 (IBM) and graphs were generated using GraphPad Prism Version 10. In all analyses, the statistical significance of the result is indicated with *p* values.

## Supplementary Information

Below is the link to the electronic supplementary material.


Supplementary Material 1


## Data Availability

Datasets generated during the current study are available from the corresponding author on reasonable request.

## References

[1] Shankar-Hari, M. et al. Developing a new definition and assessing new clinical criteria for septic shock. *JAMA* **315**, 775 (2016). 26903336 10.1001/jama.2016.0289 PMC4910392

[2] Daniels, R., Nutbeam, T. & Berry, E. Sepsis in adults. *BMJ Best Pract.* https://bestpractice.bmj.com/topics/en-gb/3000098 (2025).

[3] Fleischmann, C. et al. Assessment of global incidence and mortality of hospital-treated sepsis. Current estimates and limitations. *Am. J. Respir. Crit. Care Med.* **193**, 259–272 (2016). 26414292 10.1164/rccm.201504-0781OC

[4] Deen, J. et al. Community-acquired bacterial bloodstream infections in developing countries in south and southeast Asia: A systematic review. *Lancet Infect. Dis.* **12**, 480–487 (2012). 22632186 10.1016/S1473-3099(12)70028-2

[5] Limmathurotsakul, D. Causes and outcomes of sepsis in southeast Asia: A multinational multicentre cross-sectional study. *Lancet Glob. Health* **5**, e157–e167 (2017). 28104185 10.1016/S2214-109X(17)30007-4 PMC5332551

[6] GBD 2019 Antimicrobial Resistance Collaborators. Global mortality associated with 33 bacterial pathogens in 2019: A systematic analysis for the Global Burden of Disease Study 2019. *Lancet* **400**, 2221–2248 (2022). 10.1016/S0140-6736(22)02185-7 PMC9763654 36423648

[7] Limmathurotsakul, D. et al. Predicted global distribution of *Burkholderia pseudomallei* and burden of melioidosis. *Nat. Microbiol.* **1**, 15008 (2016). 27571754 10.1038/nmicrobiol.2015.8

[8] Limmathurotsakul, D. et al. Increasing incidence of human melioidosis in northeast Thailand. *Am. Soc. Trop. Med. Hyg.* **82**, 1113–1117 (2010). 10.4269/ajtmh.2010.10-0038 PMC2877420 20519609

[9] Meumann, E. M., Limmathurotsakul, D., Dunachie, S. J., Wiersinga, W. J. & Currie, B. J. *Burkholderia pseudomallei* and melioidosis. *Nat. Rev. Microbiol.* **22**, 155–169 (2024). 37794173 10.1038/s41579-023-00972-5

[10] Wiersinga, W. J. et al. Melioidosis. *Nat. Rev. Dis. Primers* **4**, 17107 (2018). 29388572 10.1038/nrdp.2017.107 PMC6456913

[11] Jenjaroen, K. et al. T-cell responses are associated with survival in acute melioidosis patients. *PLoS Negl. Trop. Dis.* **9**, e0004152 (2015). 26495852 10.1371/journal.pntd.0004152 PMC4619742

[12] Kronsteiner, B. et al. Diabetes alters immune response patterns to acute melioidosis in humans. *Eur. J. Immunol.* **49**, 1092–1106 (2019). 31032897 10.1002/eji.201848037 PMC6618312

[13] Preechanukul, A. et al. Identification and function of a novel human memory-like NK cell population expressing CD160 in melioidosis. *iScience* **26**, 107234 (2023). 37520720 10.1016/j.isci.2023.107234 PMC10372747

[14] Kasetthat, T. et al. Early Activation of iNKT cells increased survival time of BALB/c mice in a murine model of melioidosis. *Infect. Immun.* **90**, e0026822 (2022). 10.1128/iai.00268-22 PMC9753712 36374098

[15] Wright, S. W. et al. γδ T cells mediate protection against neutrophil-associated lung inflammation in pulmonary melioidosis. *Am. J. Respir. Cell Mol. Biol.* **71**, 546–558 (2024). 38935886 10.1165/rcmb.2024-0072OC PMC11568474

[16] Porcelli, S., Yockey, C. E., Brenner, M. B. & Balk, S. P. Analysis of T cell antigen receptor (TCR) expression by human peripheral blood CD4-8- alpha/beta T cells demonstrates preferential use of several V beta genes and an invariant TCR alpha chain. *J. Exp. Med.* **178**, 1–16 (1993). 8391057 10.1084/jem.178.1.1 PMC2191070

[17] Provine, N. M. & Klenerman, P. MAIT cells in health and disease. *Annu. Rev. Immunol.* **38**, 203–228 (2020). 31986071 10.1146/annurev-immunol-080719-015428

[18] Kurioka, A., Walker, L. J., Klenerman, P. & Willberg, C. B. MAIT cells: New guardians of the liver. *Clin. Transl. Immunol.* **5**, e98 (2016). 10.1038/cti.2016.51 PMC5007630 27588203

[19] Kjer-Nielsen, L. et al. MR1 presents microbial vitamin B metabolites to MAIT cells. *Nature* **491**, 717–723 (2012). 23051753 10.1038/nature11605

[20] Reantragoon, R., Boonpattanaporn, N., Corbett, A. J. & McCluskey, J. Mucosal-associated invariant T cells in clinical diseases. *Asian Pac. J. Allergy Immunol.* **34**, 3–10 (2016). 26994620

[21] Kjer-Nielsen, L. et al. An overview on the identification of MAIT cell antigens. *Immunol. Cell Biol.* **96**, 573–587 (2018). 29656544 10.1111/imcb.12057

[22] Ussher, J. E. et al. CD161++CD8+ T cells, including the MAIT cell subset, are specifically activated by IL-12+IL-18 in a TCR-independent manner. *Eur. J. Immunol.* **44**, 195–203 (2014). 24019201 10.1002/eji.201343509 PMC3947164

[23] Georgel, P., Radosavljevic, M., Macquin, C. & Bahram, S. The non-conventional MHC class I MR1 molecule controls infection by *Klebsiella pneumoniae* in mice. *Mol. Immunol.* **48**, 769–775 (2011). 21190736 10.1016/j.molimm.2010.12.002

[24] Meierovics, A., Yankelevich, W.-J. C. & Cowley, S. C. MAIT cells are critical for optimal mucosal immune responses during in vivo pulmonary bacterial infection. *Proc. Natl. Acad. Sci.* **110**, E3119-28 (2013). 10.1073/pnas.1302799110 PMC3746930 23898209

[25] Wang, H. et al. MAIT cells protect against pulmonary Legionella longbeachae infection. *Nat. Commun.* **9**, 3350 (2018). 30135490 10.1038/s41467-018-05202-8 PMC6105587

[26] Trivedi, S. et al. Mucosal-associated invariant T (MAIT) cells mediate protective host responses in sepsis. *Elife* **9**, e55615 (2020). 10.7554/eLife.55615 PMC7679140 33164745

[27] Grimaldi, D. et al. Specific MAIT cell behaviour among innate-like T lymphocytes in critically ill patients with severe infections. *Intensive Care Med.* **40**, 192–201 (2014). 24322275 10.1007/s00134-013-3163-x

[28] Kurioka, A. et al. MAIT cells are licensed through granzyme exchange to kill bacterially sensitized targets. *Mucosal Immunol.* **8**, 429–440 (2015). 25269706 10.1038/mi.2014.81 PMC4288950

[29] Ussher, J. E., Klenerman, P. & Willberg, C. B. Mucosal-associated invariant T-cells: New players in anti-bacterial immunity. *Front. Immunol.* **5**, 450 (2014). 10.3389/fimmu.2014.00450 PMC4189401 25339949

[30] Bernal, I. et al. *Clostridioides difficile* activates human mucosal-associated invariant T cells. *Front. Microbiol.* **9**, 2532 (2018). 10.3389/fmicb.2018.02532 PMC6209678 30410474

[31] Ussher, J. E. et al. TLR signaling in human antigen-presenting cells regulates MR1-dependent activation of MAIT cells. *Eur. J. Immunol.* **46**, 1600–1614 (2016). 27105778 10.1002/eji.201545969 PMC5297987

[32] Bzdyl, N. M., Moran, C. L., Bendo, J. & Sarkar-Tyson, M. Pathogenicity and virulence of *Burkholderia pseudomallei*. *Virulence* **13**, 1945–1965 (2022). 36271712 10.1080/21505594.2022.2139063 PMC9635556

[33] Tuanyok, A. et al. The genetic and molecular basis of O-antigenic diversity in *Burkholderia pseudomallei* lipopolysaccharide. *PLoS Negl. Trop. Dis.* **6**, e1453 (2012). 22235357 10.1371/journal.pntd.0001453 PMC3250505

[34] Huo, S. et al. Characterization of *Burkholderia pseudomallei* O antigens in different clinical strains. *Int. J. Biol. Macromol.* **225**, 795–808 (2023). 36402383 10.1016/j.ijbiomac.2022.11.143

[35] Khongpraphan, S. et al. Differentiation in pyroptosis induction by *Burkholderia pseudomallei* and *Burkholderia thailandensis* in primary human monocytes, a possible cause of sepsis in acute melioidosis patients. *PLoS Negl. Trop. Dis.* **18**, e0012368 (2024). 39042701 10.1371/journal.pntd.0012368 PMC11296640

[36] Lovelace-Macon, L. et al. Flagellin-modulated inflammasome pathways characterize the human alveolar macrophage response to *Burkholderia pseudomallei*, a lung-tropic pathogen. *Infect. Immun.* **92**, e0006024 (2024). 10.1128/iai.00060-24 PMC11075458 38619302

[37] Lichtenegger, S. et al. Burkholderia pseudomallei triggers canonical inflammasome activation in a human primary macrophage-based infection model. *PLoS Negl. Trop. Dis.* **14**, e0008840 (2020). 33137811 10.1371/journal.pntd.0008840 PMC7605897

[38] Kim, J.-C. et al. Deficiencies of circulating mucosal-associated invariant T cells and natural killer T cells in patients with acute cholecystitis. *J. Korean Med. Sci.* **30**, 606 (2015). 25931792 10.3346/jkms.2015.30.5.606 PMC4414645

[39] Kwon, Y.-S. et al. Mucosal-associated invariant T cells are numerically and functionally deficient in patients with mycobacterial infection and reflect disease activity. *Tuberculosis* **95**, 267–274 (2015). 25837440 10.1016/j.tube.2015.03.004

[40] Malka-Ruimy, C. et al. Mucosal-associated invariant T cell levels are reduced in the peripheral blood and lungs of children with active pulmonary tuberculosis. *Front. Immunol.* **10**, 206 (2019). 10.3389/fimmu.2019.00206 PMC6396712 30853958

[41] Kang, S.-J. et al. Activation, impaired tumor necrosis factor-α production, and deficiency of circulating mucosal-associated invariant T cells in patients with scrub typhus. *PLoS Negl. Trop. Dis.* **10**, e0004832 (2016). 27463223 10.1371/journal.pntd.0004832 PMC4963088

[42] Salerno-Goncalves, R. et al. Challenge of humans with wild-type *Salmonella enterica* Serovar Typhi Elicits changes in the activation and homing characteristics of mucosal-associated invariant T cells. *Front. Immunol.* **8**, 398 (2017). 10.3389/fimmu.2017.00398 PMC5382150 28428786

[43] Hotchkiss, R. S., Monneret, G. & Payen, D. Sepsis-induced immunosuppression: from cellular dysfunctions to immunotherapy. *Nat. Rev. Immunol.* **13**, 862–874 (2013). 24232462 10.1038/nri3552 PMC4077177

[44] Zheng, Y. et al. MAIT cell activation and recruitment in inflammation and tissue damage in acute appendicitis. *Sci. Adv.* **10**, eadn6331 (2024). 10.1126/sciadv.adn6331 PMC11168461 38865451

[45] Dias, J. et al. The CD4-CD8- MAIT cell subpopulation is a functionally distinct subset developmentally related to the main CD8+ MAIT cell pool. *Proc. Natl. Acad. Sci.* **115**, E11513–E11522 (2018). 10.1073/pnas.1812273115 PMC6298106 30442667

[46] Wong, E. B., Ndung’u, T. & Kasprowicz, V. O. The role of mucosal-associated invariant T cells in infectious diseases. *Immunology* **150**, 45–54 (2017). 27633333 10.1111/imm.12673 PMC5341498

[47] Wiersinga, W. J. et al. Endogenous interleukin-18 improves the early antimicrobial host response in severe melioidosis. *Infect. Immun.* **75**, 3739–3746 (2007). 17517876 10.1128/IAI.00080-07 PMC1951987

[48] Ceballos-Olvera, I., Sahoo, M., Miller, M. A., del Barrio, L. & Re, F. Inflammasome-dependent pyroptosis and IL-18 protect against burkholderia pseudomallei lung infection while IL-1β Is deleterious. *PLoS Pathog.* **7**, e1002452 (2011). 22241982 10.1371/journal.ppat.1002452 PMC3248555

[49] Dunachie, S. & Chamnan, P. The double burden of diabetes and global infection in low and middle-income countries. *Trans. R. Soc. Trop. Med. Hyg.* **113**, 56–64 (2019). 30517697 10.1093/trstmh/try124 PMC6364794

[50] Magalhaes, I. et al. Mucosal-associated invariant T cell alterations in obese and type 2 diabetic patients. *J. Clin. Investig.* **125**, 1752–1762 (2015). 25751065 10.1172/JCI78941 PMC4396481

[51] Carolan, E. et al. Altered distribution and increased IL-17 production by mucosal-associated invariant T cells in adult and childhood obesity. *J. Immunol.* **194**, 5775–5780 (2015). 25980010 10.4049/jimmunol.1402945

[52] Pisarska, M. M., Dunne, M. R., O’Shea, D. & Hogan, A. E. Interleukin-17 producing mucosal associated invariant T cells—emerging players in chronic inflammatory diseases?. *Eur. J. Immunol.* **50**, 1098–1108 (2020). 32617963 10.1002/eji.202048645

[53] Kaukonen, K.-M., Bailey, M., Pilcher, D., Cooper, D. J. & Bellomo, R. Systemic inflammatory response syndrome criteria in defining severe sepsis. *N. Engl. J. Med.* **372**, 1629–1638 (2015). 25776936 10.1056/NEJMoa1415236

