# Supplementary material for: Mucosal-associated invariant T (MAIT) cells are reduced and dysfunctional in acute melioidosis
Source: Sci Rep. 2026 Jun 15;16:27368. doi: 10.1038/s41598-026-57890-8 (PMC13530224; doi:10.1038/s41598-026-57890-8)
Supplement: Supplementary file 1 [file 41598_2026_57890_MOESM1_ESM.pdf]

Supporting Information

Mucosal-associated invariant T (MAIT) cells are reduced and dysfunctional in acute melioidosis

Fazle Rabbi Chowdhury[1,2,3,4], Martha Zewdie[1,2], Priyanka Abraham[1,2,4], Srija Moulik[1,2], Hadiza Adebowale[1,2], Jennifer Hill[1,2], Md Robed Amin[5,6], Lovely Barai[7], Md Rokibul Hasan[7], Ayako Kurioka[1], Patpong Rongkard[1,2], Direk Limmathurotsakul[4,8], Nicholas PJ Day[4], Parinya Chamnan[9], Paul Klenerman[1,10,11,12], Chris B Willberg[1], Barbara Kronsteiner[1,2,*], Susanna J Dunachie[1,2,4,10,*]

[1] Peter Medawar Building for Pathogen Research, University of Oxford, Oxford, United Kingdom.

[2] NDM Centre for Global Health Research, University of Oxford, Oxford, United Kingdom.

[3] Department of Internal Medicine, Bangladesh Medical University, Dhaka, Bangladesh

[4] Mahidol-Oxford Tropical Medicine Research Unit, Mahidol University, Bangkok, Thailand.

[5]Department of Medicine, Dhaka Medical College, Dhaka, Bangladesh

[6]Directorate General of Health Services, Dhaka, Bangladesh

[7]Department of Microbiology, BIRDEM General Hospital, Dhaka, Bangladesh

[8]Department of Tropical Hygiene, Faculty of Tropical Medicine, Mahidol University, Bangkok, Thailand

[9]Cardiometabolic Research Group, Sunpasitthiprasong Hospital, Ubon Ratchathani, Thailand

[10]Oxford University Hospitals NHS Foundation Trust, Oxford, UK

[11]Translational Gastroenterology Unit, University of Oxford, UK

[12]NIHR Oxford Biomedical Research Centre, University of Oxford, Oxford, UK

*These authors contributed equally

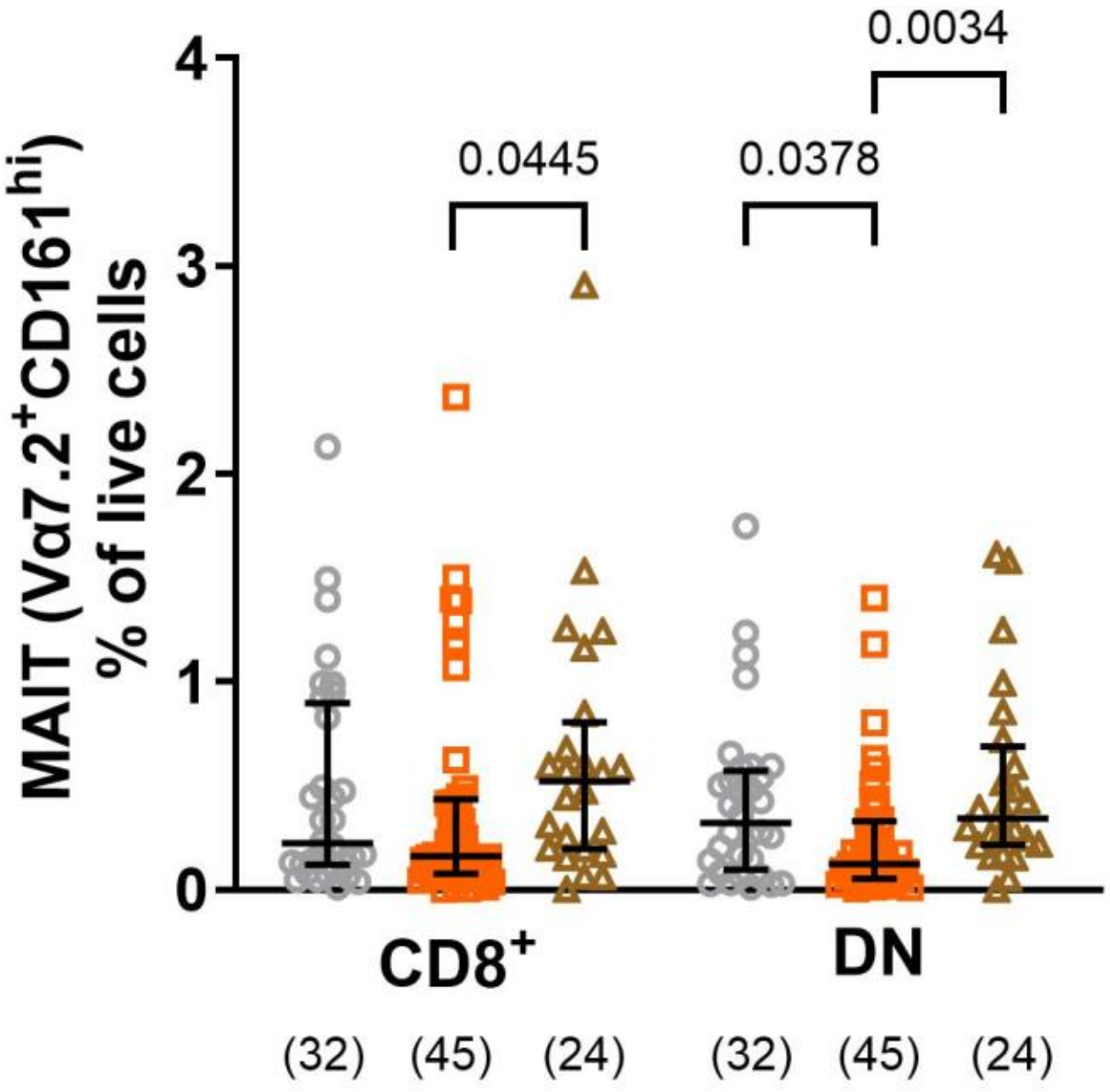

Supplementary Figure 1. MAIT cell frequency is reduced in acute melioidosis. Frequency of CD8+ and double negative (DN) MAIT cells within live cells was compared between controls, acute melioidosis patients and those who had recovered from melioidosis (1 year post admission). Grey circles: controls, orange squares: acute patients, brown triangles: recovered patients. The number of biological replicates per group is given in brackets under graphs. A two-tailed p value of <0.05 was considered statistically significant. Exact p values <0.1 are displayed on graphs. The data are displayed as median and interquartile range. The three groups were compared using Kruskal Wallis test followed by Dunn's multiple comparison.

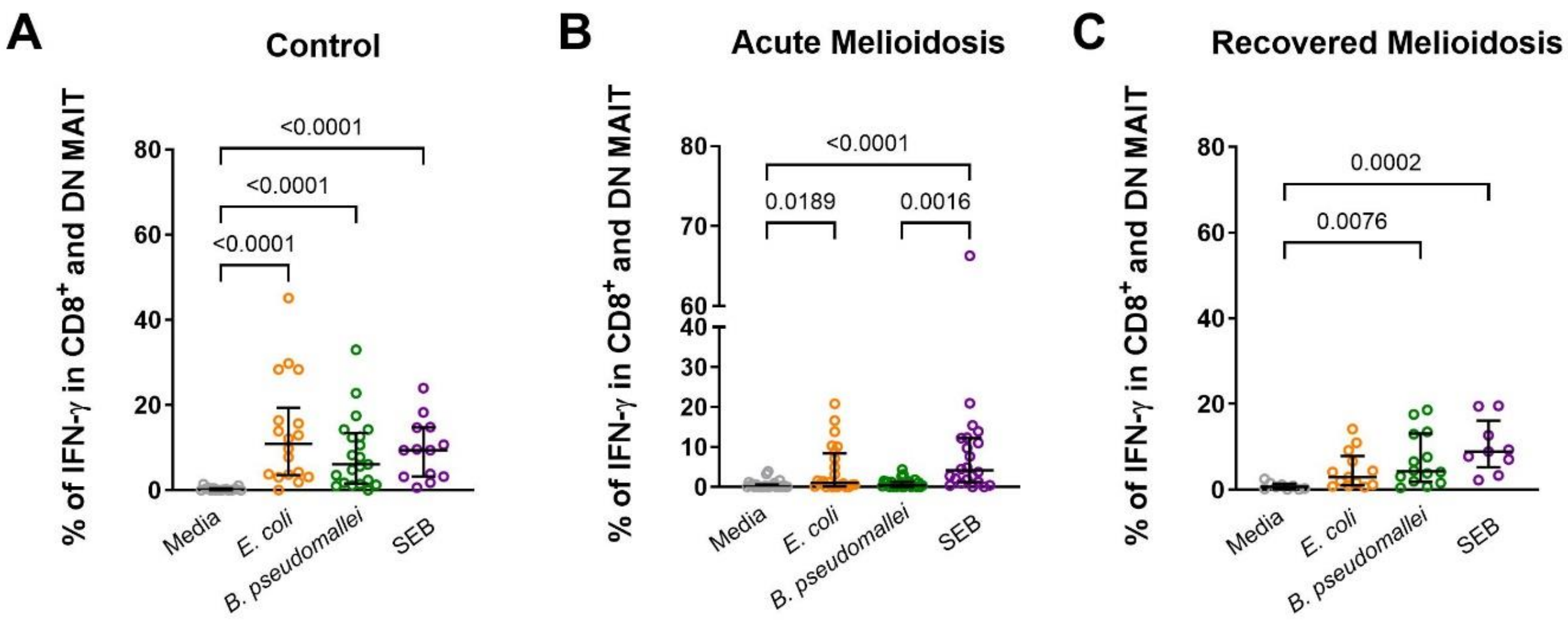

Supplementary Figure 2. MAIT cell function in melioidosis. Expression of IFN-γ in MAIT cells (CD8+ and DN) upon *in vitro* stimulation with fixed *E. coli*, *B. pseudomallei* (BP), staphylococcal enterotoxin B (SEB) and media was assessed in (A) healthy controls, (B) acute melioidosis patients and (C) those who had recovered from melioidosis (1 year post admission). A two-tailed p value of <0.05 was considered statistically significant. Exact p values <0.1 are displayed on graphs. The data are displayed as median and interquartile range. The four treatment groups were compared using Kruskal Wallis test followed by Dunn's multiple comparison.

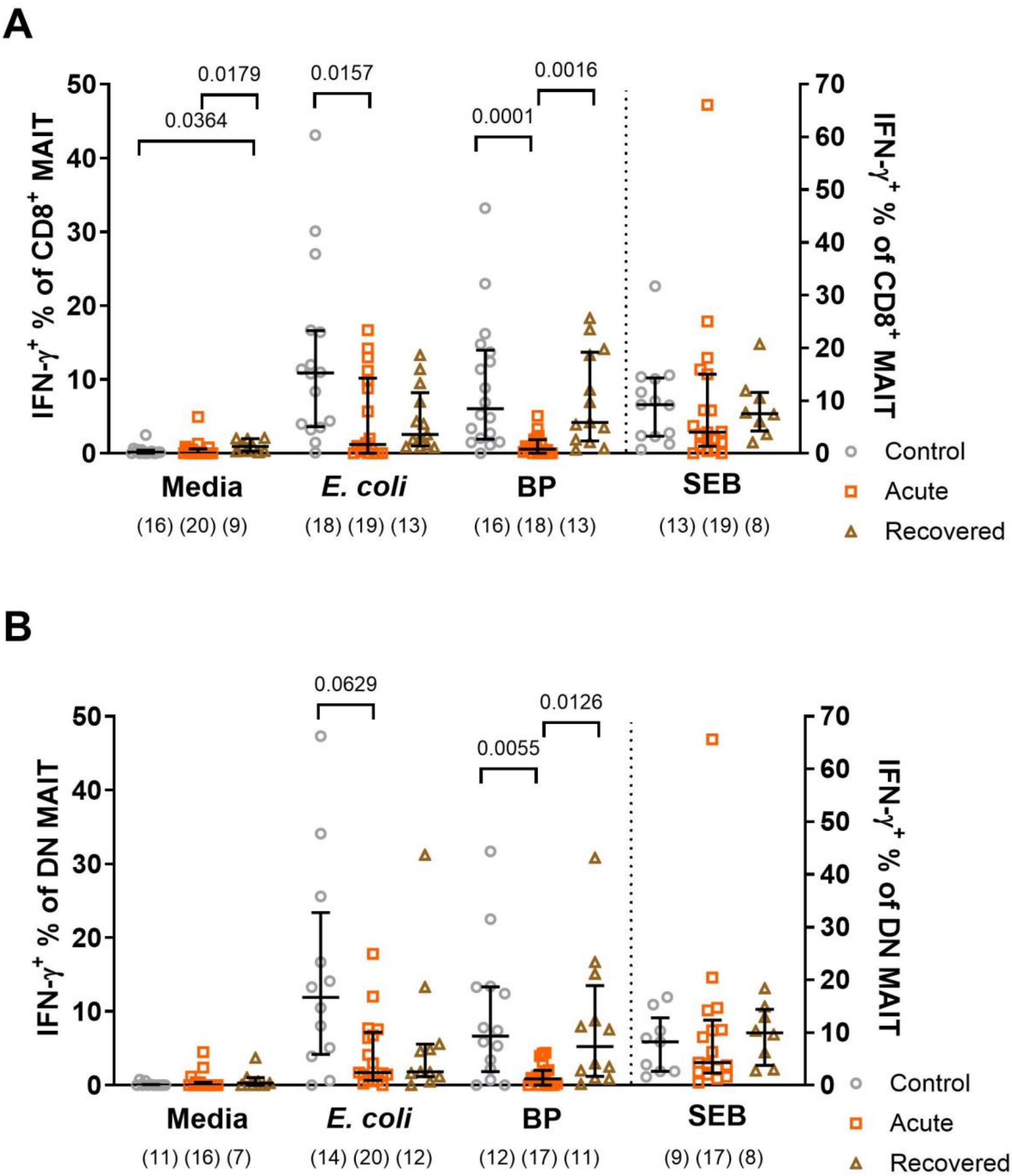

Supplementary Figure 3. CD8+ and DN MAIT cell function in melioidosis. Expression of IFN-γ in (A) CD8+ MAIT cells and (B) DN MAIT cells upon *in vitro* stimulation with fixed *E. coli*, *B. pseudomallei* (BP), staphylococcal enterotoxin B (SEB) and media was compared between controls, acute melioidosis patients and those who had recovered from melioidosis (1 year post admission). Grey circles: controls, orange squares: acute patients, brown triangles: recovered patients. The number of biological replicates per group is given in brackets under

graphs. A two-tailed p value of <0.05 was considered statistically significant. Exact p values <0.1 are displayed on graphs. The data are displayed as median and interquartile range. The three groups were compared using Kruskal Wallis test followed by Dunn's multiple comparison. DM= diabetes mellitus.

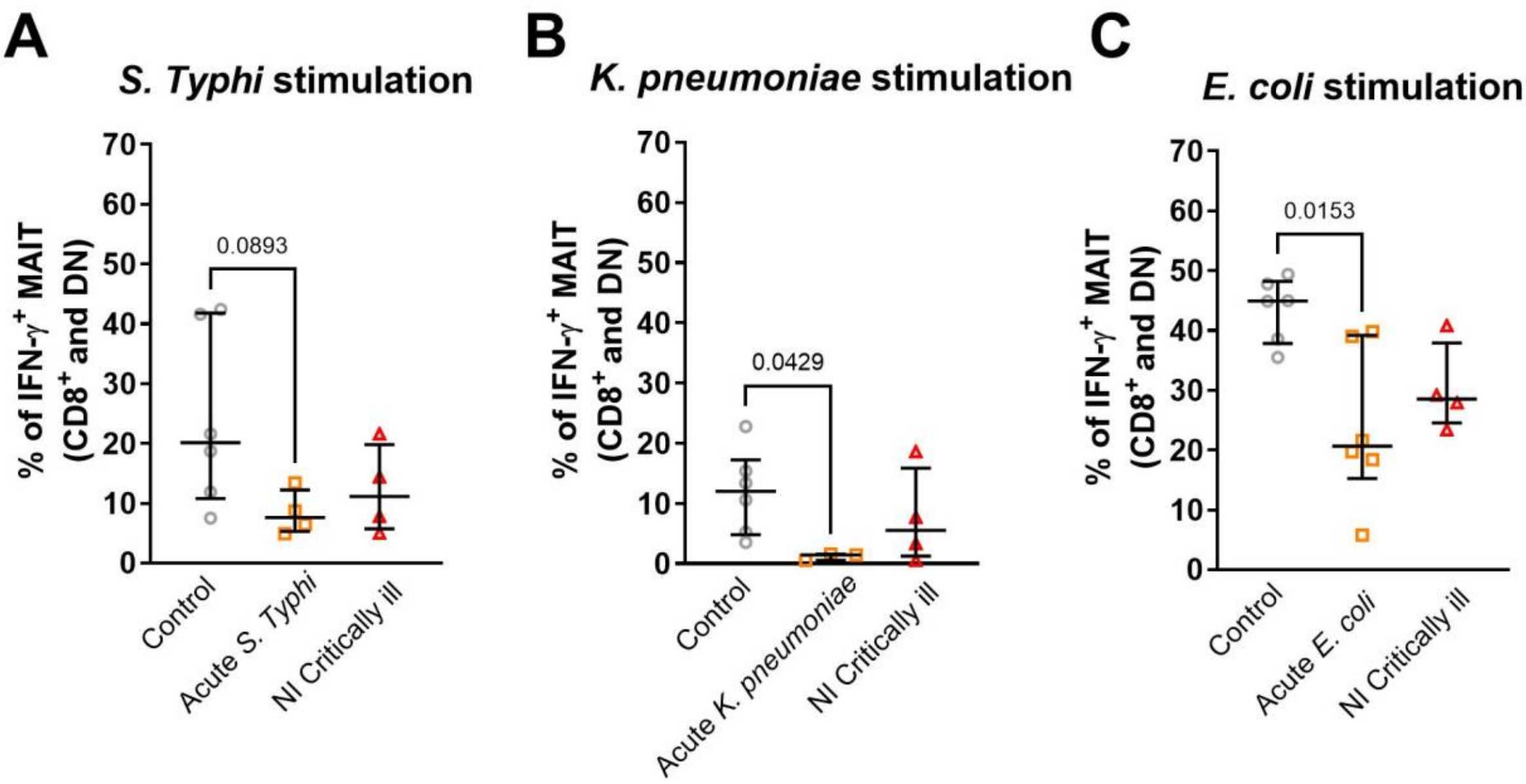

Supplementary Figure 4. Reduced MAIT function is common in Gram-negative sepsis. IFN-γ expression in the combined CD8+ and DN MAIT cell population was compared between patients with acute sepsis caused by different Gram-negative bacteria (*E. coli* n=6, *S.* Typhi n=4, *K. pneumoniae* n=3), healthy controls (n=6) and non-infectious (NI) critically ill cases (n=4) upon stimulation with the respective causative bacteria (fixed). A two-tailed p value of <0.05 was considered statistically significant. Exact p values <0.1 are displayed on graphs. The data are displayed as median and interquartile range. Three or more groups were compared using Kruskal Wallis test followed by Dunn's multiple comparison.

Supplementary Table 1. Details of antibodies used for flow cytometry experiments

Marker	Fluorochrome	Clone	Manufacturer	Panel	Figure
CD3	PE-Cy5	UCHT1	Biolegend	Phenotyping and MAIT cytokine secretion assay	Figure 2-4
CD3	eFluor450	OKT3	eBioscience	MAIT cytokine secretion assay	Figure 1
CD4	V450	L200	BD Biosciences	Phenotyping and MAIT cytokine secretion assay	Figure 2-4
CD4	VioGreen	VIT4	Miltenyi Biotec	MAIT cytokine secretion assay	Figure 1
CD8	BV510	RPA-T8	Biolegend	Phenotyping and MAIT cytokine secretion assay	Figure 2-4
CD8	PE-Vio770	BW135/80	Miltenyi Biotec	MAIT cytokine secretion assay	Figure 1
CD161	PE	191B8	Miltenyi Biotec	Phenotyping and MAIT	Figure 1-4

				cytokine secretion assay	
TCR Vα7.2	APC	3C10	Biolegend	Phenotyping and MAIT cytokine secretion assay	Figure 1-4
CD69	PE-Vio770	FN50	Miltenyi Biotec	Phenotyping	Figure 2-4
GzmB	FITC	GB11	Biolegend	Phenotyping	Figure 2-4
IFN-γ	PE-Cy7	4S.B3	Biolegend	MAIT cytokine secretion assay	Figure 2-4
IFN-γ	FITC	45-15	Miltenyi Biotec	MAIT cytokine secretion assay	Figure 1
TNF	FITC	MAB11	eBioscience	MAIT cytokine secretion assay	Figure 2-4
TNF	PerCP-Cy5.5	MAB11	Biolegend	MAIT cytokine secretion assay	Figure 1

Supplementary Table 2. Demographic and clinical characteristics of acute melioidosis patients who survived and died within 28-days of admission

	Survived<br>N=30	Died<br>N=25	p-value*
**Age, years**			0.13
Median age	63	57	
Age range	44-80	33-84	
Interquartile range	53-70	50-62	
**Sex, n (%)**			0.3
Female	7 (23%)	9 (36%)	
Male	23 (77%)	16 (64%)	
**DM Status, n (%)**			0.55
DM	18 (60%)	13 (52%)	
Non-DM	12 (40%)	12 (48%)	
**Bacteremic, n (%)**	15 (50%)	24 (96%)	<0.001

*Pearson Chi-Squared test; Mann-Whitney U test; Fisher's exact test

Supplementary Table 3. Demographic and clinical characteristics of acute melioidosis patients with and without DM

	DM N=31	Non-DM N=24	p-value*
**Age**			0.021
Median age	57	63	
Age range	43-80	33-84	
Interquartile range	49-62	57-71	
**Sex, n (%)**			0.074
Female	12 (39%)	4 (17%)	
Male	19 (61%)	20 (83%)	
**28-Day Mortality, n (%)**			0.55
Survived	18 (58%)	12 (50%)	
Died	13 (42%)	12 (50%)	
**History of renal impairment, n (%)**	6 (19%)	5 (31%)	0.47
**HbA1c**			<0.001
Median (Q1-Q3)	11.10 (8.6-12.6)	5.8 (5.3- 6.2)	

*Pearson Chi-Squared test; Mann Whitney U test; Fisher's exact test
